# EXERCISE EFFECTS ON WELL-BEING ON CANADIAN WOMEN OVER THE AGE OF 65

**DOI:** 10.1093/geroni/igz038.453

**Published:** 2019-11-08

**Authors:** Holly A Bardutz, Constance Corley

**Affiliations:** 1 University of Regina, Regina, Saskatchewan, Canada; 2 Fielding Graduate University, Santa Barbara, California, United States

## Abstract

Two groups of Canadian women, over the age of 65, were interviewed (n = 20) in a study focused on brain health. The first group (n = 12) regularly attended exercise classes and met the exercise standards of the Canadian Society of Exercise Physiologists for the age group over 65 years for at least six months. Women in the comparison group (n = 8) had been taking adult education classes twice a week or more for at least six months. Thematic coding was used to analyze the results. Both groups reported benefits from their participation in their respective groups. However, the results show that the group who exercised regularly consistently reported improved mood, increased mental alertness, a better ability to handle stress, less pain, and improved sleep. These factors were not reported by the non-exercise group, which did benefit by gaining new knowledge, making new friends and feeling good because they were learning new things. This study suggests that Canadian women over the age of 65 who have been exercising regularly report many of the effects of exercise on the brain that are beneficial to their well-being. They did not specifically mention the new brain cells being made (neurogenesis) nor did they note neuronal rewiring (neuroplasticity), however they did self-report some psychological benefits that the Comparison Group did not report, as noted above. This research has implications for both practice and research.

